# Atypical vessels as an early sign of intracardiac myxoma?

**DOI:** 10.1186/1476-7120-2-13

**Published:** 2004-08-13

**Authors:** Hans-Peter Dübel, Fabian Knebel, Volker Gliech, Wolfgang Konertz, Wolfgang Rutsch, Gert Baumann, Adrian Constantin Borges

**Affiliations:** 1 Medical Clinic for Cardiology, Angiology, Pulmology, Charité Campus Mitte, University medicine Berlin, Germany; 2 Director of the Clinic of Cardiovascular Surgery, Charité Campus Mitte, University Medicine Berlin, Germany

**Keywords:** Myxoma, Coronary Artery Disease

## Abstract

We report on a woman with previously unknown left atrial myxoma, who underwent percutaneous coronary intervention. 45 months after the initial coronary angiography, echocardiography demonstrated a large atrial myxoma, which was not seen echocardiographically before. The retrospective analysis of the pre-intervention coronary angiography revealed atypical vessels in the atrial septum, which are interpreted as early signs of myxoma.

## Background

Primary cardiac tumors are rare disorders, with an incidence of 0.02% at autopsy. Three quarters of the primary tumors of the heart are benign, half of which are myxomas [1].

As noninvasive cardiac imaging becomes widely available, with increasing resolution provided by echocardiography, computed tomography and magnetic resonance imaging, cardiac tumors are being diagnosed more often. Angiography, apart from its preoperative role to rule out concomitant coronary artery disease, is rarely needed for the diagnostic work-up of cardiac tumors.

This report describes the delayed presentation of a left atrial myxoma which was not depicted in an initial coronary angiography performed 51 months earlier in a woman with chest pain.

## Case Report

**Figure 1:**
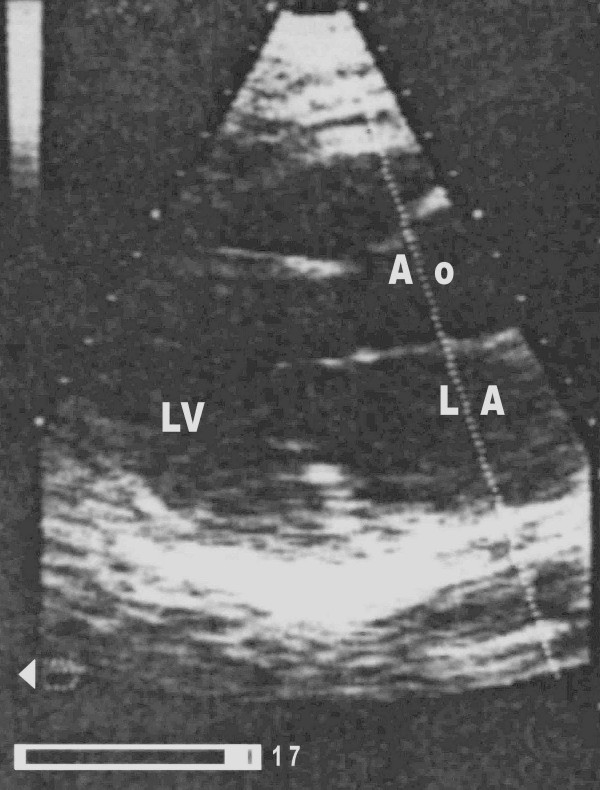
Stress echocardiography: long axis view (baseline). No sign of myxoma in left chamber or in left atrium. LA = left atrium, LV = left ventricle, Ao = Ascending aorta (with kind permission of Dr. Herbst, Potsdam)

A 62-year-old woman with known metabolic syndrome was referred to our clinic to exclude coronary artery disease invasively. She has been experiencing chest pain for four months which has not increased in frequency or duration since it started. She denied pain at rest, nocturnal pain, difficulty breathing, or palpitations. An echocardiographic stress examination revealed significant ischemia with anteroseptal hypokinesia. Cardiac chambers were morphologically normal. The "baseline" echocardiography and stress echo was unsuspicious of a left atrial myxoma (Fig. 1).

Coronary angiography revealed coronary artery disease with a stenosis of the proximal left anterior descending coronary artery (LAD) near the left main stem. A stent was implanted in the proximal LAD with no residual stenosis. The ventricle was morphologically normal.

**Figure 2:**
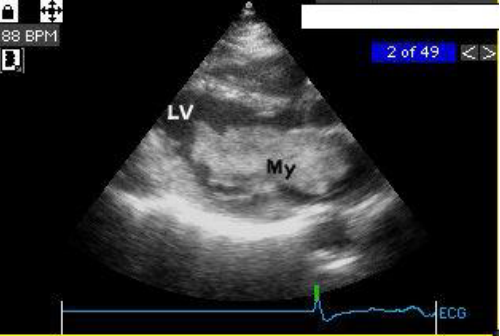
Echocardiography (45 months) long axis view. Myxoma in the left atrium prolapsing into the left ventricle. LV = left ventricle, My = myxoma

A coronary angiography performed 13 months after stent implantation showed no re-stenosis and a normal left ventricle. No echocardiogram was performed at this time 32 months after the control coronary angiography, the patient was readmitted because of increasing dyspnoea and palpitations. A transthoracic echocardiography disclosed a big (70 × 30 mm) mass in the left atrium attached to the interatrial septum. The tumor prolapsed into the left ventricle obstructing the mitral valve orifice (Fig 2). The mean pressure gradient across the mitral valve was 8 mm Hg.

**Figure 5:**
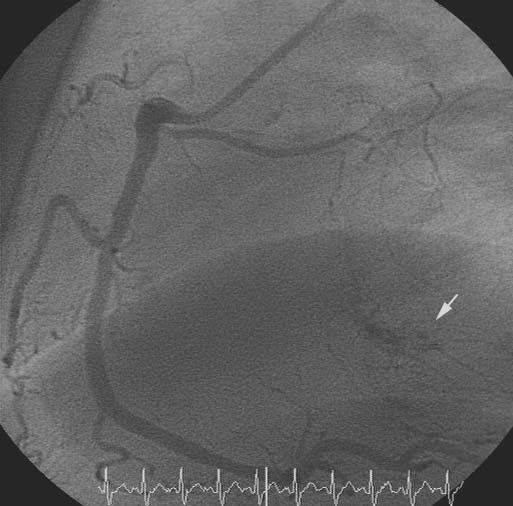
Right coronary artery (RCA) in 60 degree LAO position (45 months, pre-operative coronary angiography). White arrow = atypical vessels in the interatrial septum

A subsequent coronary angiography and LV and RV catheterization detected a mean diastolic pressure gradient of 12 mm Hg between the pulmonary capillary wedge and the left ventricular enddiastolic pressure, and no re-stenosis of the LAD stent. The angiography was notable for a large area with small atypical, tortuous vessels in the region of the interatrial septum. These vessels were shown to originate from branches of the right coronary artery (RCA) and the circumflex coronary artery (RCX). (Fig. 5)

Surgery was promptly performed and the tumor was successfully excised. Histology confirmed the diagnosis of a cardiac myxoma.

**Figure 3:**
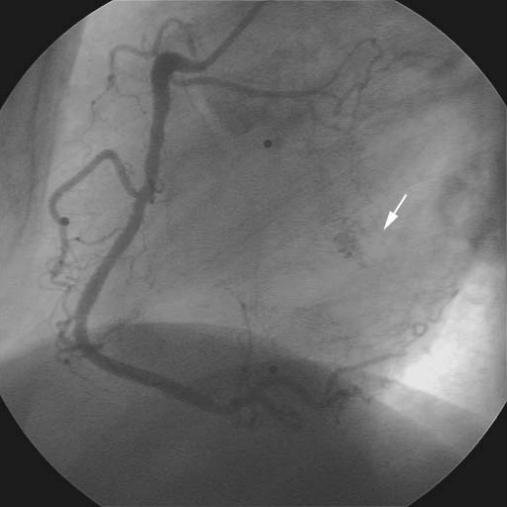
Right coronary artery (baseline) in 90 degree LAO projection. White arrow = atypical vessels in the interatrial septum

**Figure 4:**
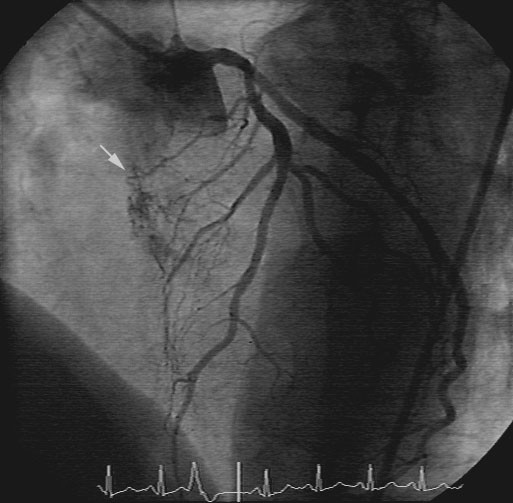
Left coronary artery (13 months) in 45 degree LAO and 30 degree CRAN projection. White arrow = atypical vessels in the interatrial septum

A retrospective analysis of the initial coronary angiographies (baseline, 13 and 45 months) disclosed the atypical vessels in a small area of interatrial septum (Fig. 3, 4, 5).

## Discussion

Myxomas are benign but potentially dangerous with disturbance of rhythm, peripheral embolization or mechanical valvular obstruction, of the atrial or ventricular cavity [2,3].

The site, mobility, and size of the myxoma determine the clinical course. Some authors found no correlation between the size of the tumor and the clinical picture [4], others reported symptoms with left atrial myxomas weighting more than 70 g [5]. The rate of growth of myxomas is not exactly known [5,6]. An increase in size of 1.8 – 5.8 cm/year and in weight of up to 14 g/year was reported [7,8]. The myxoma of our patient reached 70 × 30 mm before being symptomatic.

Myxomas presenting with systemic embolism or intracavitary obstruction can be easily detected non-invasively. The early depiction of small intracardiac tumors by means of angiography relies on the detection of atypical vessels supplied by branches of the left or right coronary artery. Our case demonstrates that this early angiographic sign is difficult to find. However, vascular malformations are not pathognomonic for myxomas [9] and stromal tumors, such as myxomas, have generally poor blood supply, hence a coronary angiographic finding with a neo-vascularized or highly vascularized intracardiac area is more suggestive for other type of cardiac neoplasm. A cluster of small, tortuous and dilated vessels is also seen in old and organised thrombi, haemangiomas and venous malfomations [10]. The size of malformation has no relation to the size of the tumor [11].

It has been reported in the literature that myxomas can be induced by radiation [12,13]. Between the first presentation to our clinic and the diagnosis of myxoma, the patient underwent two coronary angiograms including stenting, with a cumulative radiological exposure of about 30 mSv (corresponding to at least 1500 chest X-rays). To our knowledge, the patient did not undergo any other relevant radiation exposure in the past.

In the present case, a retrospective analysis of the patient angiographies disclosed the atypical vessels which were initially overseen. These vessels could have probably been interpreted as an early sign of myxoma.

## Author's contributions

HPD and FK have written the manuscript and have equally contributed to this publication. HPD, VG and WR have performed the coronary angiographies. WK has performed cardiac surgery. HPD, ACB, FK and GB participated in the design and coordination of the final manuscript. All authors have read and approved the final manuscript.

## References

[B1] Reynen K (1996). Frequency of primary tumors of the heart. Am J Cardiol.

[B2] Colucci WS, Schoen FJ, Braunwald E, Braunwald E (1997). Primary tumors of the heart. Heart Disease. A Textbook of Cardiovascular Medicine.

[B3] Shapiro LM (2001). Cardiac tumours: diagnosis and management. Heart.

[B4] Vassiliadis N, Vassiliadis K, Karkavelas G (1997). Sudden death due to cardiac myxoma. Med Sci Law.

[B5] Roberts WC (1997). Primary and secondary neoplasms of the heart. Am J Cardiol.

[B6] Reynen K (1993). Benigne Tumoren des Herzens [Benign tumors of the heart]. Z Kardiol.

[B7] Malekzadeh S, Roberts WC (1989). Growth rate of left atrial myxoma. Am J Cardiol.

[B8] Pochis WT, Wingo MW, Cinquegrani MP, Sagar KB (1991). Echocardiographic demonstration of rapid growth of a left atrial myxoma. Am Heart J.

[B9] Van Cleemput J, Daenen W, De Geest H (1993). Coronary angiography in cardiac myxoma: findings in 19 consecutive cases and review of literature. Cathet Cardiovasc Diagn.

[B10] Fueredi GA, Knechtges TE, Czarnecki DJ (1989). Coronary angiography in atrial myxoma: Findings in nine cases. AJR Am J Roentgenol.

[B11] Chow WH, Chow TC, Tai YT, Yip ASB, Cheung KL (1991). Angiographic visualization of „tumor vascularity" in atrial myxoma. Eur Heart J.

[B12] Daoud J, Ben Salah H, Kammoun W, Ghorbel A, Frikha M, Jlidi R, Besbes M, Drira MM, Maalej M (2000). Radiation-induced glioblastoma and myxoma after treatment for undifferentiated carcinoma of the naspharynx. Cancer Radiother.

[B13] Douniau R, Dambrain R (1970). Effect of ionizing radiation on the teeth and maxillofacial bone structure during development. Report of a case of myxoma. Acta Stomatol Belg.

